# Contrast-enhanced spectral mammographic findings of phyllodes tumor of the breast

**DOI:** 10.1186/s43055-022-00789-x

**Published:** 2022-05-13

**Authors:** Sebnem Orguc, Seda Mavili, Çağdaş Rıza Açar, Hasan Aydede, Ali Rıza Kandiloğlu

**Affiliations:** 1 Depermant of Radiology, Manisa Celal Bayar University Medical School, Manisa, Turkey; 2 Department of Pathology, Manisa Celal Bayar University Medical School, Manisa, Turkey; 3 Department of Surgery, Manisa Celal Bayar University Medical School, Manisa, Turkey

**Keywords:** Phyllodes tumors, Contrast-enhanced spectral mammographic (CESM), Breast

## Abstract

**Background:**

Phyllodes tumors of breast are rare fibroepithelial neoplasms. They have similar radiological findings with fibroadenomas. While fibroadenomas are benign lesions, phyllodes tumors may have malignant potential. Therefore, any imaging findings to differentiate fibroadenoma from phyllodes tumor are valuable.

**Case presentation:**

A 51-year-old female patient was admitted to our clinic with the complaint of a palpable mass. Tru-Cut biopsy resulted as phyllodes tumor, and excision was recommended. However, the patient neglected herself during the COVID-19 pandemic, and 20 months later, she presented with a huge and complex mass. On CESM imaging, cystic areas and clefts were identified. The case was diagnosed as borderline phyllodes tumor.

**Conclusions:**

Although MRI findings of phyllodes tumor are well known and reported many times, there is no information about CESM findings of this tumor in literature. Bubbly appearance on CESM is useful finding in the diagnosis of phyllodes tumor of breast.

## Background

Phyllodes tumors are rare, rapidly growing tumors which account for about 1% of all breast neoplasms [1]. They usually present with a palpable mass in middle-aged women.

Contrast-enhanced spectral mammography (CESM) is a valuable tool for evaluating breast masses. It combines iodinated contrast agent with digital X-ray mammography obtained with dual energy. This combination evaluates the morphology of the mass with a high spatial resolution, as well as adding the power of contrast material in the assessment of functional properties of the breast mass.

Phyllodes is derived from the Latin word Phyllodium which means ‘leaf-like’ based on a gross pathological description of a leafy, bulky, cystic and fleshy tumor of the breast [2]. They are biphasic fibroepithelial neoplasms composed of stromal and epithelial components [3]. Abundant cellular stroma in typical leaf-like architecture differentiates them from fibroadenomas and other sarcomas [4]. World Health Organisation subclassifies phyllodes tumors as benign, borderline or malignant depending on the degree of overgrowth, cellular atypia and rate of mitosis [5]. Approximately, 20–30% of resected phyllodes tumors are malignant [6].

Wide surgical excision with negative surgical margins (≥ 1 cm) without axillary lymph node dissection is the standard treatment method. Risk of local recurrence is as high as 65% in borderline and malignant subtypes and ranges from 5 to 30% in benign phyllodes tumors [7].

Phyllodes tumor and fibroadenoma have similar radiological findings. Fibroadenoma and phyllodes tumor may appear as mass lesions with distinct borders on conventional mammography. Tumor size over 3 cm, poorly demarcated borders and microlobulated architecture favor the diagnosis of phyllodes tumor. Cystic spaces which can be demonstrated by US and T2W sequences on breast MRI can be used for the differentiation of phyllodes tumor from fibroadenoma. Post-gadolinium-enhanced MRI findings of phyllodes tumor are reported in the literature; intense enhancement with no washout represents type 1 kinetic curve [8, 9].

CESM is also a post-contrast imaging modality demonstrating the functional properties of the breast tumor similar to breast MRI. To our knowledge, this is the first report of enhancement properties of phyllodes tumor as defined by CESM. Excellent spatial resolution of mammography combined with contrast enhancement demonstrates the well-defined borders as well as cystic spaces with rim enhancement within the tumor. This appearance is highly suggestive of phyllodes tumor. We also propose that more vivid enhancement may be an indicator for malignant potential. Further case series and studies are needed to support CESM findings phyllodes tumors.

## Case presentation

A 51-year-old female presented with a palpable mass in her left breast in July 2019. There was no personal or family history of breast cancer. The hypoechoic mass with lobulated contours located at the periareolar area of the outer quadrant was measured 28 × 20 × 212 mm in diameter by ultrasonography. The mass had vascularity on color Doppler imaging. It had no microcalcifications, cystic or necrotic areas. Tru-Cut biopsy under ultrasound guidance resulted as phyllodes tumor, and excision of the lesion was recommended.

**Fig. 1:**
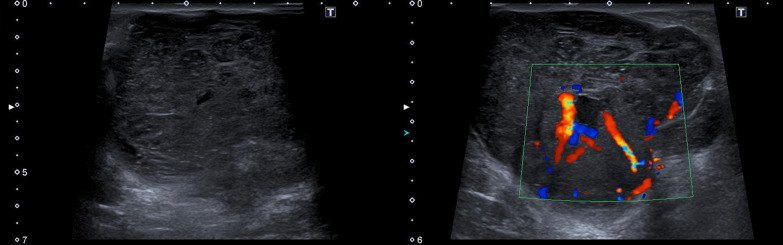
**A** Ultrasonographic and **B** Color Doppler examination demonstrates hypoechoic areas which represent cystic areas and increased vascularization of the tumor

**Fig. 2:**
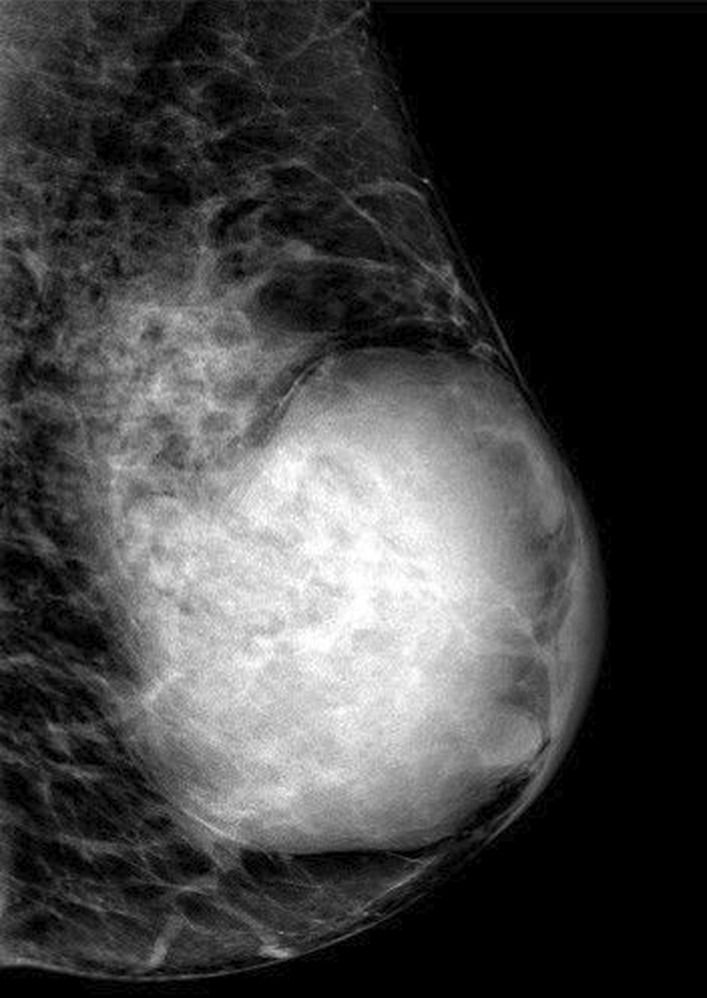
Low-energy mammogram in medio-lateral oblique (MLO) position of the left breast demonstrates giant breast mass with no calcifications

**Fig. 3:**
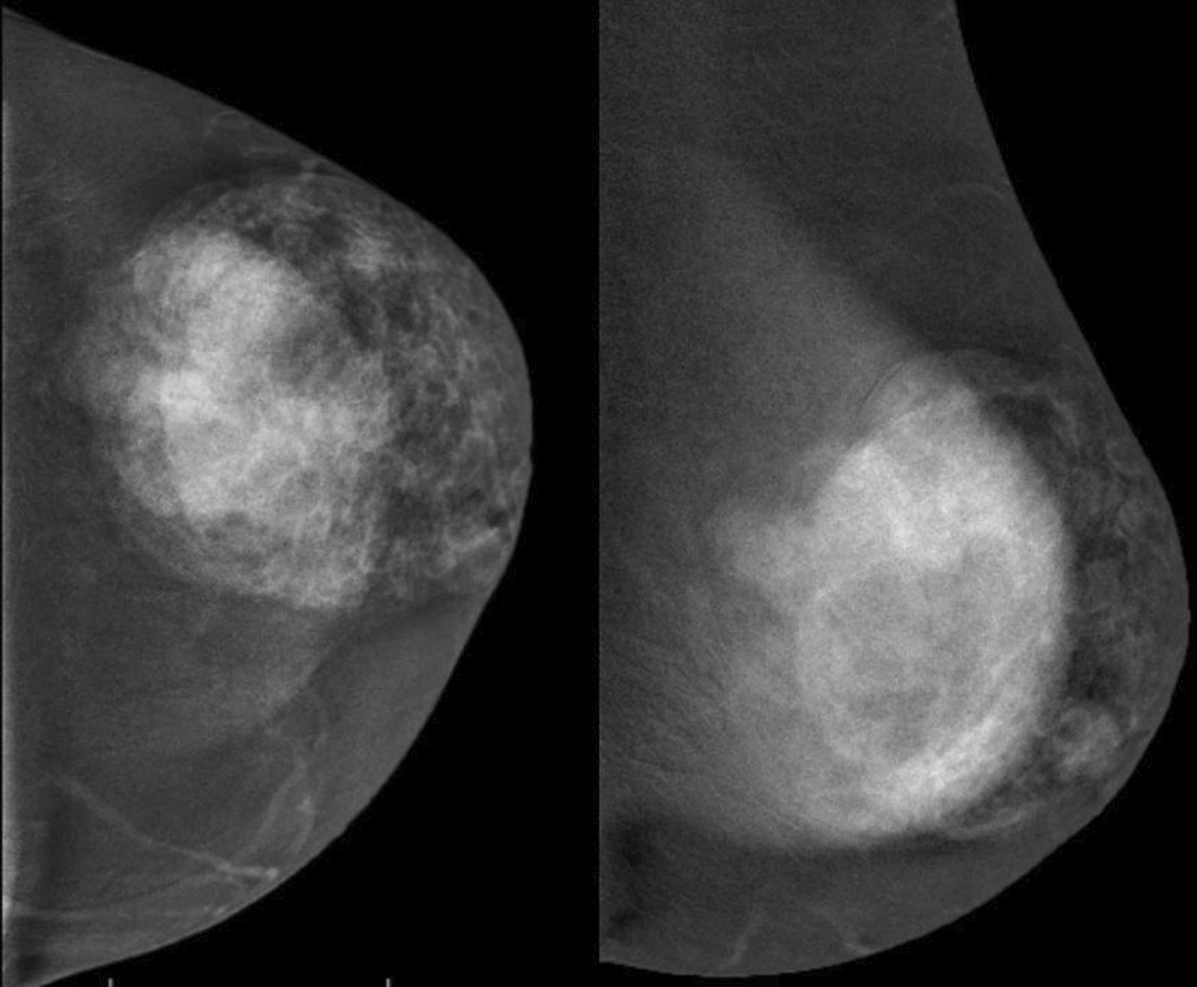
Contrast-enhanced spectral mammograms of the left breast in **A** Craniocaudal (CC), **B** Medio-lateral oblique (MLO) positions demonstrate the well-defined giant breast mass with macrolobulations. The solid components of the mass surrounding the cystic branching clefts enhanced moderately with administration of İV iodinated contrast material. Note that background parenchymal enhancement of the breast tissue increases in the MLO image which is obtained later in the series

Unfortunately, the patient neglected herself during the COVID-19 pandemic and she was admitted to our clinic once again in March 2021, approximately 20 months later with a huge mass. The size of the breast increased dramatically, and lesion could even be seen easily by visual inspection. The lesion measured 8.5 × 8 × 7.5 cm in diameter and was more vascular when compared with the previous examination. Ultrasonographic and color Doppler imaging examination demonstrated increased vascularization and also revealed new cystic areas (Fig. 1A, B). On low-energy image mammography, the lesion was well circumscribed and had no calcification (Fig. 2). On contrast-enhanced spectral mammography, solid parts of the lobulated lesion enhanced vividly with contrast. There were multiple, well-defined, non-enhancing areas representing cystic changes. Cystic areas and clefts outlined by the enhancing solid component were eccentric in location. These findings created a bubbly appearance which was a clue for phyllodes tumor (Fig. 3A, B). We demonstrated this contrast enhancement pattern with CESM for the first time in literature. The superb spatial resolution of CESM enabled demonstration of both morphologic and metabolic properties of the phyllodes tumor in one stop shop. We performed a second Tru-Cut biopsy from this lesion prior to excision to rule out possible interval malignant transformation.

**Fig. 4:**
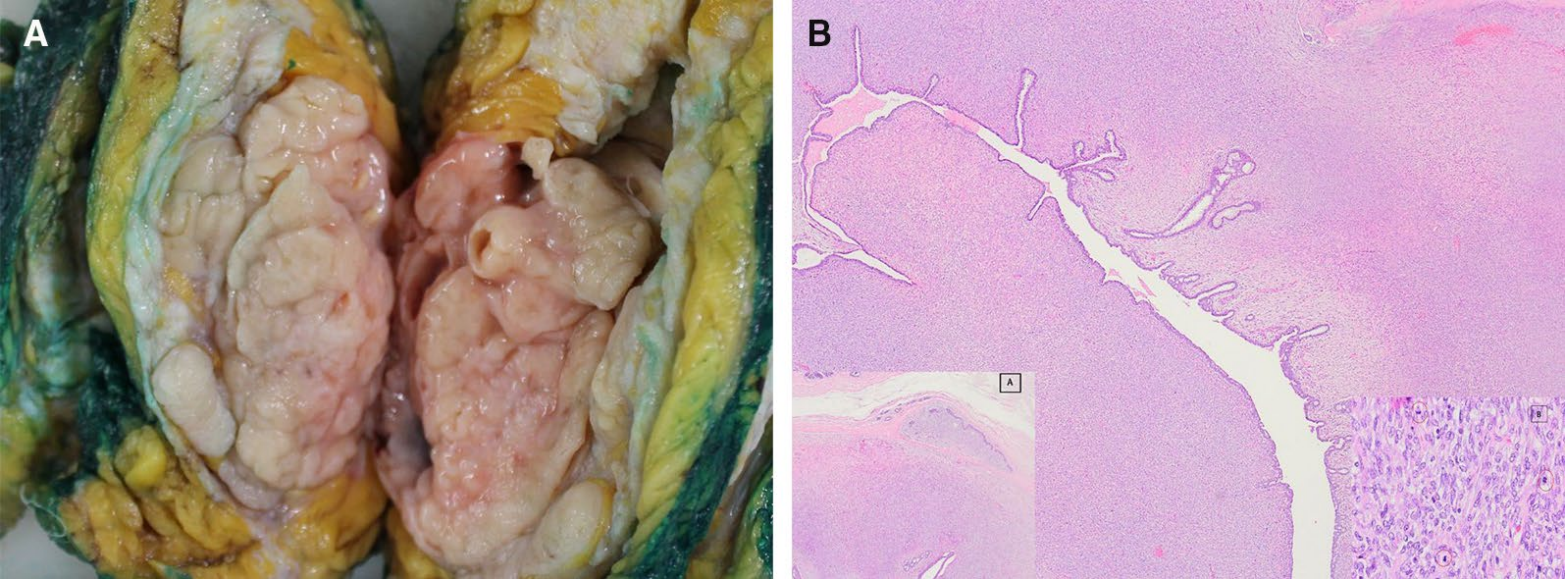
**A** Macroscopic appearance of cut surface of the tumor composing of solid, firm, tan to pink tissue. In most areas tumor is well circumscribed measuring 8.2 cm. Note that whorled pattern and clefts can be seen. **B** (H&E; × 20 and H&E; × 400). Microscopic appearance of the tumor which is composed predominantly of cellular mesenchymal areas along with pronounced clefts lined by bland epithelium. Note tongue-like focal infiltrative border (inset **A**). Epithelium was surrounded by stromal cells showing mild/moderate atypia and numerous mitoses (circles, inset **B**)

On macroscopic examination of the mastectomy material, a tumor measuring 8.2 × 8 × 7.5 cm was observed. On cut surface, it was relatively well-circumscribed, with vague nodular pattern, containing clefts and small cystic areas (Fig. 4A). Upon microscopic examination, in most areas tumor had well-circumscribed borders; however, in a few foci it was noticed that it had created extensions toward the normal breast parenchyma as tongues. The tumor was comprised by long, some branching, cleft-like spaces and duct lined by an inner epithelial and outer myoepithelial cell layer. They were surrounded by a rather cellular stroma containing cells showing mild or moderate atypia and 13 mitoses were observed at 10 high power fields in the densest areas (Fig. 4B). Ki-67 immunohistochemistry revealed a proliferative index of 40 percent. No tumor was seen at the surgical margins. The case was diagnosed as borderline phyllodes tumor due to the absence of stromal overgrowth, marked stromal cell atypia, intensive infiltrative border and/or malignant heterologous elements despite the presence of prominent mitotic activity.

## Conclusions

This is the first case report demonstrating enhancement of the solid components of phyllodes tumor which creates a unique bubbly appearance on CESM, suggesting the diagnosis in rapidly growing breast masses.

## Data Availability

All data generated or analyzed during this study are included in this article and are available at Manisa Celal Bayar University, Hafsa Sultan Hospital, Department of Radiology, Manisa, Turkey.

## Abbreviations

CESM: Contrast-enhanced spectral mammographic
MRI: Magnetic resonance imaging
T2W: T2 weighted
US: Ultrasonography
